# Evaluation of the Effects of Agro Pesticides Use on Liver and Kidney Function in Farmers from Buea, Cameroon

**DOI:** 10.1155/2020/2305764

**Published:** 2020-01-29

**Authors:** Faustin Pascal Tsagué Manfo, Sharon Asukia Mboe, Edouard Akono Nantia, Ferdinand Ngoula, Phélix Bruno Telefo, Paul Fewou Moundipa, Fidelis Cho-Ngwa

**Affiliations:** ^1^Department of Biochemistry and Molecular Biology, Faculty of Science, University of Buea, PO Box 63 Buea, Cameroon; ^2^Department of Biochemistry, Faculty of Science, University of Bamenda, PO Box 39 Bambili, Cameroon; ^3^Department of Animal Sciences, Faculty of Agronomy and Agricultural Sciences, University of Dschang, P.O. Box 188 Dschang, Cameroon; ^4^Department of Biochemistry, Laboratory of Medicinal Plant Biochemistry, Food Science, and Nutrition, Faculty of Science, University of Dschang, Dschang, Cameroon; ^5^Laboratory of Pharmacology and Toxicology, Department of Biochemistry, Faculty of Science, University of Yaoundé I, P.O. Box 812 Yaoundé, Cameroon; ^6^Laboratory for Drugs and Molecular Diagnostics Research (ANDI Centre of Excellence for Onchocerciasis Drug Research), Biotechnology Unit, University of Buea, Buea, Cameroon

## Abstract

Agro pesticides are increasingly used worldwide to increase crop production. However, health hazards resulting from human exposure to these chemicals, especially from agricultural areas of developing countries have been a growing concern. The objective of this study was to evaluate the impact of occupational exposure to agro pesticides on the health of farmers in the Buea subdivision, which is one of the major agrarian areas in Cameroon. The study was transversal and involved 90 participants including 58 farmers using pesticides and a reference population of 32 men not involved in occupational use of agro pesticides. The participants were interviewed on agro pesticide use and their health status. Thereafter, blood samples were collected from the participants and used for the assessment of biochemical markers of the liver (alanine aminotransferase and aspartate aminotransferase) and the kidney (creatinine and uric acid) function. Results revealed that farmers frequently used insecticides, fungicides, and herbicides in their farming activities. Farmers reported several acute health symptoms related to pesticides use with the common ones being skin rash, eye irritation, and face burn. When compared to the reference population, the farmers showed significantly elevated (*p* < 0.01) alanine aminotransferase activity. However, other parameters investigated were not affected significantly. These results suggested that farmers were exposed to 3 different classes of agro pesticides, which induced eye and skin affections. Pesticides exposure resulted in alterations of the liver function hence the increased serum alanine aminotransferase activity. Therefore, there is a need to sensitize the farmers on toxicity and liver alteration potential of agro pesticides and the importance of appropriate protective equipment that may minimize exposure.

## 1. Introduction

Pesticides are chemicals deliberately introduced into the environment to kill or control pests (insects, rodents, birds, weeds, fungi) that interfere with crop production, storage of food grains, and eliminate vectors implicated in diseases transmission [1, 2]. Pesticides are mainly used by farmers to boost productivity in agriculture, a critical activity for human welfare and economic growth in developing countries including Cameroon. Agriculture accounts for a large share of the gross domestic product and employs a major proportion of the labour force in rural areas of Cameroon where majority of people live on small-scale farms****[3–5]. Agriculture is the primary occupation of the rural population in the southwest region of Cameroon, where several industrial farms are located. Small-scale farmers from the region produce substantial amount of food and cash crops such as cereals, vegetables, tubers/root crops, cocoa and perennials [6, 7]. The tropical climate of this agrarian region, characterized by high temperatures, and humid conditions, favours pests proliferation and plant diseases thereby promoting high use of pesticides by the farmers to enable increased crop production. These include insecticides and fungicides, which are more heavily applied on tropical crops than crops in temperate regions [8].

Agro pesticides are not appropriately used by farmers, as illegal utilization of some banned agrochemicals (lindane, malathion, and dimethoate) has been reported recently in the southwest region of Cameroon [9]. Indiscriminate pesticide usage and accumulation of obsolete pesticides have also been observed in Cameroon and other developing countries [9–11]. Application of pesticide without adequate protective tools, was reported in small-scale farmers from Djutitsa and Buea in Cameroon [5, 6], and may lead to the impairment of farmers' health. Although the amount of pesticide generally used in developed countries supersedes that of the developing countries, death rate due to these chemicals is higher in developing countries [12, 13]. This is probably related to handling conditions of the chemicals, as several studies have demonstrated correlation between the indiscriminate and inappropriate use of pesticides and adverse health effects on humans, especially farmers [14, 15].

Pesticides are generally taken up by farmers through inhalation, ingestion or dermally, and distributed through the circulatory system to affect various organs. The Liver and kidneys are largely acknowledged as important organs involved in detoxification of organisms, through metabolism and excretion of the xenobiotics and related metabolites. These organs are therefore especially vulnerable to damage by xenobiotics such as pesticides [2, 16]. The nephrotoxic and hepatotoxic effects of agro pesticides have been illustrated by several investigations in laboratory animals [17, 18]. Alteration of the liver and kidney function has been reported in farmers from Palestine and India [19, 20]. Other investigations also revealed positive association between exposure to agro pesticides and liver dysfunction markers [21, 22].

Despite the frequent and increased use of agro pesticides in the Buea subdivision in Cameroon, the effects of these chemicals on the health of farmers applying the chemicals is still unclear. The present study was therefore undertaken to investigate the effects of agro pesticide exposure on the liver and kidney function of male farmers from the Buea subdivision.

## 2. Materials and Methods

### 2.1. Study Population and Questionnaire

**Figure 1:**
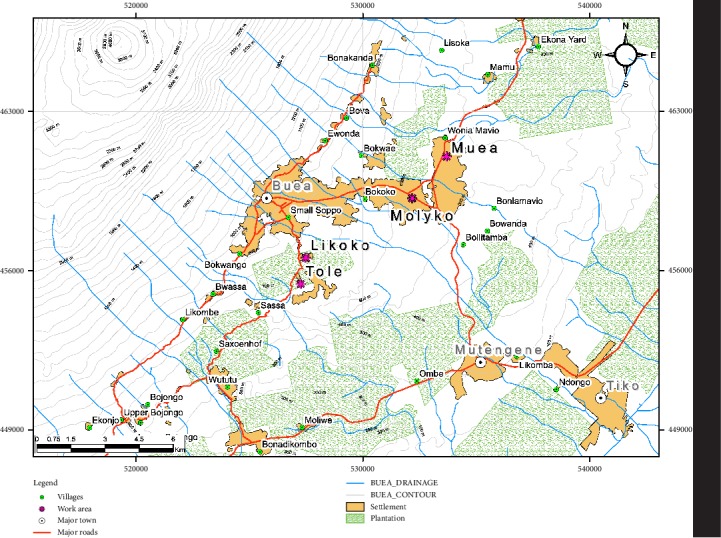
A map showing sampling locations.

This case-control study enrolled 90 male participants including 58 farmers routinely involved in spraying agro pesticides (group occupationally exposed to agro pesticides) and a reference group of 32 individuals who were not involved in occupational use of pesticides. The study was conducted in 2017, and involved participants residing in the Buea subdivision, specifically from the localities of Buea, Likoko, Muea, and Tole (Figure 1).

The inclusion criteria were as follows: male human, willing to participate, aged 20–60 years, and not under medication. Also, the participants had to be apparently healthy, i.e., no chronic diseases or liver and kidney dysfunction (diabetes, liver cirrhosis, kidney failure, and hypertension) based on interview. Using a structured questionnaire, the participants were interviewed on their socio-demographic information (age, level of education, occupation) and agro pesticides used, pesticide application devices, safety measures (disposal of pesticide wastes, use of protective gears), and symptoms related to agro pesticide use.

The study protocol was approved by the Institutional Ethics Committee for Human Research of the Faculty of Health Science of the University of Buea in Cameroon (Ref: 2016/0.53/UB/FHS/IRB), and administrative authorization obtained from the Regional Delegation for the South West region of Cameroon, Ministry of Public Health (Ref: R11/MINSANTE/SWR/RDPH/PS/068/280). All participants provided written informed consent, and were free to withdraw from the research at any time during the investigations. However, none of the participants withdrew from the study.

### 2.2. Blood Sample Collection and Storage

After an overnight fast, 5 mL of blood was collected from each participant aseptically into vacutainer tubes without anticoagulant. Samples were maintained at 4°C, and serum separated by centrifugation (3000 rpm, 15 min, 4°C) and stored at −20°C for further assessment of biochemical markers of the liver and kidney function.

### 2.3. Biochemical Assays

For biochemical assessment of the liver and kidney function, activities of alanine aminotransferase (ALT) and aspartate aminotransferase (AST), creatinine levels, and uric acid concentration were all measured in the serum samples using kits from Chronolab Systems, S.L.—Barcelona, Spain.

#### 2.3.1. Aminotransferase Enzymes

Each serum sample (100 *µ*L) was mixed with 1 mL working reagent into a 1 cm cuvette to constitute a reaction mixture. In the mixture,****aminotransferase enzymes (AST/ALT) from serum sample catalyse the transfer of an amino group from aspartate (for AST) or alanine (for ALT) to *α*-ketoglutarate forming glutamate (or pyruvate) and oxaloacetate. The oxaloacetate is reduced to malate by malate dehydrogenase and NADH. The rate of decrease in the concentration of NADH (change in absorbance per minute, ΔA/min), which is proportional to the catalytic concentration of AST/ALT in the serum sample analysed, was measured using a spectrophotometer at 340 nm, and the enzyme activity calculated using the formula: Δ*A*/min × 1750 = *U*/*L*.

#### 2.3.2. Creatinine

A mixture of****100 *µ*L of serum sample and 1 ml of alkaline solution containing picrate was prepared in a 1cm cuvette. Under these conditions, picrate reacted with creatinine to from an orange complex, whose formation rate between 30 seconds and 90 seconds (Δ*Ab*_Sample_ = Sample_30s_ − Sample_30s_) is determined spectrophotometrically at 492 nm. A standard solution of creatinine (2 mg/dl) was also analysed under the same conditions, and the corresponding change in absorbance (Δ*Ab*_Std_ = Std_90s_ − Std_30s_) was used to calculate the creatinine concentration in the serum sample as follows:

(1) $$\begin{array}{c} \text{Creatinine}\left(\mu \text{M}\right)=\left[\frac{\Delta {Ab}_{\text{Sample}}}{\Delta {Ab}_{\text{Std}}}\right]\times 2\times 88.4. \end{array}$$

#### 2.3.3. Uric Acid

Determination of serum uric acid was done using a colorimetric kit from Chronolab Systems, with slight modifications of the protocol. The Working reagent (WR) was prepared as described by the kit manufacturer, and the original assay protocol modified to suit a microplate adaptation. Briefly, 200 *µ*L of the working reagent was mixed with either 5 *µ*L serum samples (test), uric acid standard (Std, 6 mg/dl), or WR (blank), and incubated for 5 min. Upon incubation, the uric acid reacted with the components of the WR to produce a red colour, whose intensity was measured at 520 nm using an ELISA plate reader. The absorbencies of sample (*A*_Sample_), standard (*A*_Std_) and blank (*A*_blank_), was used to calculate uric acid concentration as follows:

(2) $$\begin{array}{c} \text{Uric acid}\left(\mu \text{M}\right)=\left[\frac{A_{\text{Sample}}-A_{\text{blank}}}{A_{\text{Std}}-A_{\text{blank}}}\right]\times 6\times 59.5. \end{array}$$

### 2.4. Statistical Analysis

Data obtained from this study were keyed in Microsoft Excel 2010. Descriptive statistics were performed on the qualitative data obtained from questionnaires, and the results were presented as percentages or absolute frequencies. The data were then imported into MedCalc v14.8.1.0 for analysis. Frequencies were compared using Fisher's exact test. Biochemical parameters were tested for normal distribution using Kolmogorov-Smirnov test, and averages compared between the reference group and pesticide users using either *t*-test (normally distributed data) or Welch *t*-test (assuming unequal variances), where appropriate. A *P* value <0.05 was considered statistically significant.

## 3. Results

### 3.1. General Characteristics of Participants

A total of 90 men were involved in this study including 58 farmers using pesticides and a reference population of 32 men not involved in occupational use of agro pesticides. All participants were apparently healthy, and stated that they were not suffering from kidney or liver disease. These characteristics are shown in Table 1.

As shown in Table 1, the average age of farmers and the reference population did not differ significantly (*P* = 0.3454). Conversely, there was a significant difference in the educational level (*P* < 0.001). A majority of the farmers (60.3%) had primary school as the highest educational attainment, while the reference population mainly comprised men who had reached the university level (71.9%). Also, consumption of stimulants such as alcohol, coffee/tea, and smoking of cigarettes, did not differ significantly between the reference population and the group of agro pesticide users.

### 3.2. Agro Pesticide Use, Protective Equipment and Reported Symptoms of Exposure

About half of the farmer's population had used pesticides for ≤5 years while 30% were involved in handling/application of the agrochemicals for more than 10 years (Table 2).

Awareness on pesticide toxicity differed significantly between the 2 studied groups, with an overall 40.6% (25.0 + 15.6) men from the reference population stating that pesticides have major harmful or fatal effects on humans, vs. 12.1% (10.3 + 1.7) agro pesticide users only (*P* < 0.01). Farmers mainly stored or kept agrochemicals at home (82.8% farmers) upon purchase. After using the chemicals, majority of the farmers (91.4%) disposed empty containers at the opened field in the farm, while others buried them in the ground (3.4%), burned (3.4%) or threw into the river (1.7%). The farmers generally purchased agro pesticides locally from retailers, and applied the chemicals on cultures using mainly knapsack sprayers as equipment. The sprayers were generally cleaned/washed in the farm (96.6% farmers).

Data on agro pesticides used by the farmers are presented in Table 3. The farmers used a total of 24 pesticide active ingredients including 5 herbicides, 10 insecticides, and 9 fungicides. It should be noted that each farmer used 7−12 active ingredients, comprising at least one from each pesticide class. However, fungicides were the most frequently used agrochemicals, as illustrated by the high relative frequency of this pesticide group (53.14% relative frequency to the total active ingredients). The active ingredients of the pesticides were sold under 27 formulations including 11, 9, and 7 fungicide, insecticide, and herbicide formulations, respectively. Glyphader and Roundup were the most frequently used herbicides formulation. The most used fungicide formulations were Mancostar 80WP and Trimangol 80WP, while insecticides were mainly available as Gammalin 20 and Mocap. The pesticides were generally applied on crops using knapsak sprayers (93.1% of farmers).

The use of protective equipment while applying pesticides was also investigated. It was observed that only 1.7% farmers used gloves and 3.1% used face masks. The main protective tools were long trousers and long shirts, which were mentioned by 63.8% and 60.3% of the farmers, respectively. However, these clothes were not generally made of impermeable material.

**Figure 2:**
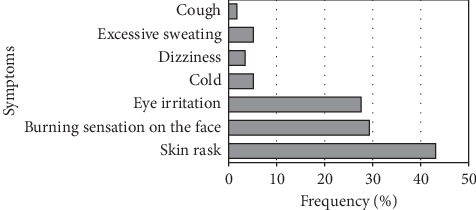
Postapplication symptoms of agropesticide exposure.

Almost all farmers, that is 86.2%, reported to have felt at least one symptom following pesticide application or handling. As shown in Figure 2, these symptoms mainly included skin rash, face burn, and eye irritation, which were each reported by at least 25% of the farmers. Four other symptoms including cold, dizziness, and excessive sweating were reported less frequently by the farmers.

### 3.3. Biochemical Markers of Liver and Kidney Function

**Figure 3:**
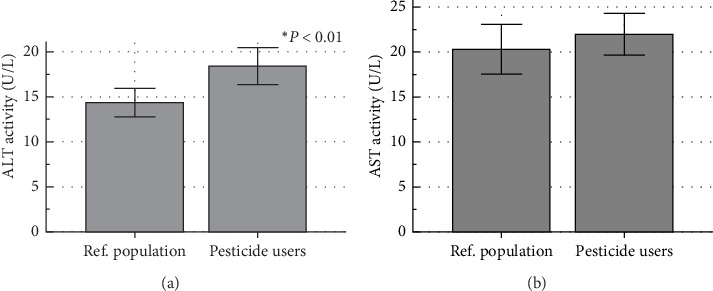
Activities of alanine aminotransferase (a) and aspartate aminotransferase (b) in a reference population and agro pesticide users. ALT: Alanine aminotransferase; AST: Aspartate aminotransferase; Ref. population: Reference population. Significant difference from the reference population at ^∗^*P* < 0.01 (Welch *t*-test).

Activities of ALT and AST were measured in serum samples from both farmers and the reference population and the results presented in Figure 3. It was observed that the average ALT activity was 28.4% elevated in farmers when compared to the reference population (*P* = 0.0022; Welch *t*-test), while AST activity remained not affected significantly.

AST/ALT ratio was not affected (1.47 ± 1.03 vs. 1.49 ± 0.52; *P* = 0.926; Welch *t*-test) between the two populations (Supplement 1). For further assessment of the effect of pesticides on ALT levels, a subanalysis of the results from farmers was conducted. Dichotomization of the data between users and nonusers of each pesticide revealed a significantly higher ALT activity in the serum of farmers using the fungicide formulation Callomil Plus (*P* = 0.0128) or its active ingredients metalaxyl (*P* = 0.0326) and copper oxide (*P* = 0.0128). The farmers who were using the insecticide formulations Mocap (or active ingredient ethoprophos) and Parastar also showed elevated ALT levels when compared to nonusers (*P* = 0.0123 and 0.030, respectively) (Supplement 2).

**Figure 4:**
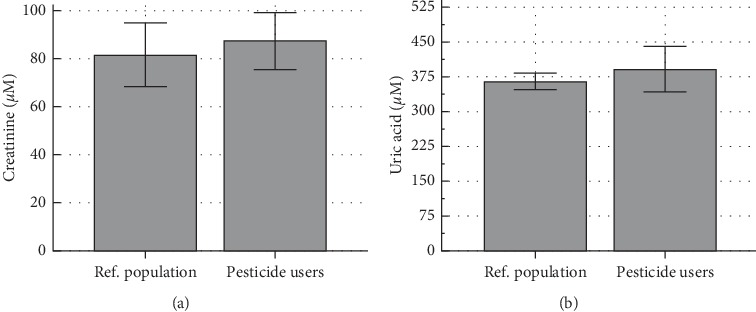
Serum levels of creatinine (a) and uric acid (b) in a reference population and agro pesticide users. Ref. population: Reference population.

Serum creatinine and uric acid, known as kidney biochemical markers, did not vary significantly between the 2 studied groups as shown in Figure 4. The ratio of these biomarkers (uric acid/creatinine ratio) was reduced by about 48% in the farmers though this difference remained not significant (11.65 ± 11.14 vs. 6.10 ± 5.12; *P* = 0.090; Welch *t*-test) (Supplement 3).

## 4. Discussion

This study was carried out to investigate the health effects of occupational exposure to pesticides among farmers from the Buea subdivision, Cameroon, focusing on pesticides handling practices by the farmers as well as pesticide effects on the liver and kidney function. A group of 58 farmers using agro pesticides and a reference population of 32 men not involved in occupational use of the agrochemicals were included in the study. The 2 study groups were similar in terms of age and consumption of stimulants such as coffee, tea, and alcohol, enabling us to reasonably compare/contrast them for the assessment of the health effects of occupational exposure to the agrochemicals.

Farmers using agro pesticides were generally less aware of the adverse effects of these chemicals on their health, and this could be justified by their lower education level when compared to the reference population. This was reflected by their habits in handling the chemicals, as up to 82.8% farmers reported keeping the pesticides at home, i.e., at the vicinity of other family members who may accidentally access the chemicals.

The farmers generally (91.4%) discarded their waste pesticide containers in the bushes or farms, instead of adopting friendly methods that may reduce distribution of the pesticide residues throughout other environmental matrices. In the context where government pesticide residue surveillance mechanisms are weak, characterized by lack of support from the authorities and unwillingness on the part of the pesticide companies to recycle empty containers [13], the dumped pesticide containers remain abandoned in the environment. Only few farmers used incineration which is recommended for some pesticides [23], though the latter method has to suit the chemical class for efficiency. Another inappropriate attitude was dumping of the containers in streams, which represent a potential danger for aquatic animals and humans.

The present study revealed a wide use of agro pesticides and also the hazardous health impacts of the substances. A total of 27 pesticide formulations with 24 active principles were identified in this study with a majority being fungicides. Predominant use of fungicides was previously reported in the South West region [9], and also among small-scale market gardening farmers from Djutitsa and Santa in Cameroon [5, 24]. This observation suggests that fungal diseases are the most proliferated and are the main threat to crops in the Buea subdivision, hence the need for farmers to use fungicides to boost agricultural productivity.

The agro pesticides sector has been liberalized in Cameroon, making it possible for the farmers to access the chemicals easily from local vendors and retailers. Because a majority of the farmers purchased their farm inputs from the local resellers, they generally have no idea if these inputs were obtained clandestinely or if they are obsolete. As a result, some active ingredients which have been banned for use in Cameroon [25], such as dimethoate and lindane, were among the agro pesticides used by the farmers. The use of these 2 active ingredients was previously reported in the Fako division by Kenko et al. [9]. Many of the farmers failed to read the pesticide label before application, hence did not follow the recommended doses. In their opinion, the manufacturers' prescription is not well appropriate to achieve effectiveness of the chemicals, so they prefer to carry out estimations. This could pose a danger to the farmers as they usually overestimate the dosage, coupled with the inexistence of, or the use of inadequate protective equipment. Indeed, exposure to pesticides occurs through the mouth, nose, and skin; and these routes could be efficiently protected by the utilization of adequate equipment such as mask, gloves, and impermeable clothing. This was not the case in the present study where a minority of farmers, less than 2%, used gloves and face masks. Most importantly, clothing was mainly a cotton wear or a permeable material, making it possible for pesticide suspension to leak /pass through the cloth and get in contact with the skin. Inappropriate and absence of protective tools during pesticide application were also reported among farmers from Djutitsa, in west Cameroon [5].

Pesticide application was mostly done manually using knapsack sprayer, commonly called “Matabi” by the farmers, and this observation corroborates with a recent report by Kenko et al. [9]. A study by Sonchieu et al. [26] also revealed a knapsack sprayer as main equipment used for pesticide application by farmers from Santa, North West region of Cameroon. Matthews et al. [3] reported that some of the sprayers used by farmers in Cameroon either had leaks or were poorly calibrated, thereby increasing dermal exposure of the farmers. Under these conditions, the farmers were more likely exposed to the agrochemicals through dermal and oral/respiratory routes, and this exposure lead to postapplication symptoms such as skin rash, face burn, and eye irritation reported by 86.2% of them. Respiratory exposure generally assumes great importance, especially for highly volatile pesticides [27]. The reported symptoms of exposure include dizziness and eye irritation or lacrimation, which generally indicate cholinergic inhibition [19, 20] by organophosphate and carbamate pesticides. Acetylcholinesterase inhibition by the pesticides induces accumulation of acetylcholine at the neuromuscular junctions and synapses, resulting in overstimulation of nicotinic and muscarinic receptors. The overstimulation of receptors results into a cholinergic crisis, characterized by the aforementioned symptoms. Other cholinergic symptoms include cramps, increased salivation, muscular weakness, paralysis, diarrhea, and blurry vision [28]. Similar observations were made by Jamal et al. [20] and Manfo et al. [5], who reported eye irritation and skin rash among pesticide applicators. The current results generally demonstrated that farmers had poor attitudes towards pesticides use. The farmers also used a wide range of agrochemicals. Each of them used 7−12 active ingredients, comprising of at least one insecticide, one fungicide, and one herbicide. Therefore, they were more likely exposed to a cocktail/mixture of the agrochemicals. These factors suggest that pesticide exposure could result in impairment of their health, and this was investigated through the assessment of their liver and kidney function.

The liver is the main metabolic organ in the body and is considered a viable defense system against environmental toxicants (xenobiotics) and metabolic toxins [29]. In the current study, its integrity was assessed through two biochemical markers, serum ALT, and AST. Results revealed a significant increase in ALT in agro pesticide users, while AST remained unchanged. ALT is a cytosolic enzyme mainly expressed by the hepatocytes, and high activity in serum samples from the farmers implies lysis of the liver cells and leakage of the enzyme into the blood, and therefore a cytotoxic effect of the agrochemicals on the liver. The increase in ALT without a significant change in AST is more indicative of liver alteration, as AST is also found in other organs such as the heart and skeletal muscle, while ALT has low concentrations in the skeletal muscle and kidney, and is chiefly produced in the hepatocytes [22, 30]. This is further sustained by exclusive localization of the ALT in cellular cytoplasm in the liver, unlike AST which is both cytosolic (20% of total activity) and mitochondrial (80% of total activity) [30]. Therefore, these changes though may not be clinically significant, indicated that frequent pesticide exposure resulted in alteration of the liver function in the farmers. The observation agrees with El-Nahhal [19], who reported liver alterations in farmers from Gaza following occupational exposure to pesticides. Other investigations from Shahzad et al. [31], Araoud et al. [21], and Jamal et al. [20] have also demonstrated increased ALT activity in individuals using pesticides. Moreover, Khan et al*., *[32] observed a positive correlation between ALT and exposure to the pesticides cypermethrin, methomyl, and imidacloprid, which were all used by the farmers. A subanalysis of the results from farmers in the current study further supports the deleterious effect of the insecticide imidacloprid, as the farmers using the imidacloprid based formulation Parastar, showed elevated ALT activity. A similar observation was made for ethoprophos, an insecticide that has been shown to induce hepatotoxicity in mice [33]. The use of the fungicide active principles metalaxyl and copper oxide was also associated with elevated ALT activity in the farmers, and corroborate previous reports on hepatotoxicity of these chemicals in rodents [34, 35]. In general, fungicides, with a cumulative frequency of 20% (as opposed to 7% for insecticides) were the most represented pesticide group associated with hepatotoxicity among farmers. Other pesticide active principles used by the farmers such as malathion and cypermethrin, though not associated with elevated ALT in the current study, were shown to induce hepatotoxicity in experimental animals [17, 36].

The kidneys, which are mainly involved in excretion of xenobiotics and related metabolites into urine, are especially vulnerable to damage by xenobiotics such as pesticides [16]. In this study, the integrity of the kidneys was assessed through serum uric acid and creatinine levels. Serum creatinine is a measure of the glomerular filtration rate and is used as an index of renal function in clinical practice [37]. Hyperuricemia has also been acknowledged as an independent predictor for kidney injury [38, 39]. Contrary to our expectations, none of the 2 renal biochemical markers were altered significantly in the farmers when compared to the reference group, suggesting integrity of the kidneys in farmers.

The nonstatistically significant variation of the uric acid /creatinine ratio (*P* = 0.090), further support nonaltered renal function, as the latter ratio was shown to positively correlate decline of estimated glomerular filtration rate [38]. Aroonvilairat et al. [40] did not also find any significant change in serum creatinine in Thai Orchid farmers occupationally exposed to agro pesticides. However, other cross-sectional studies have associated pesticide exposure with kidney dysfunction in farmers in Cameroon [41] and elsewhere [19, 20]. This has been further emphasized by Ghosh et al. [42], who elucidated a positive correlation between increased levels of pesticides in biological fluids and development of chronic kidney disease. Therefore, the pesticides should be considered as potential dangers to the kidneys despite the findings reported herein.

Although serum creatinine concentration may be regarded as the single most important laboratory parameter in routine nephrology clinical practice, assessment of additional biochemical markers such as Cystatin C, might have contributed for further evaluation of the kidney function. Consideration of the body mass index of study participants could have been useful, as muscle mass has been shown to influence serum creatinine levels [43, 44]. Other shortcomings worth mentioning include assessment of some inflammatory biomarkers and genes related to hepatotoxicity, which would have contributed for better delineation of pesticide—induced hepatotoxicity [45].

## 5. Conclusion

Overall, our findings suggested that farmers were exposed to 3 different classes of agro pesticides, which induced the eye and skin affections. Pesticide exposure resulted in alterations of the liver function hence the increased serum alanine aminotransferase activity. Therefore, there is a need to sensitize the farmers on toxicity and liver alteration potential of agro pesticides and the importance of appropriate protective equipment that may minimize exposure.

## Figures and Tables

**Table 1:** General characteristics of the study population.

Parameter	Reference group	Pesticide user	Total	*P* value
Sample size	32	58	90	

Age	33.4 ± 7.4	34.7 ± 6.0	/	0.3454 (*T*-test)

*Education level*				
Has not been to school	2 (6.3%)	1(1.7%)	3 (3.3%)	*P* < 0.0001 (Chi-squared test)
Primary school	2(6.3%)	35 (60.3%)	37 (41.1%)	
Secondary school	5(15.6%)	14 (24.1%)	19 (21.1%)	
University	23(71.9%)	8(13.8%)	31(34.4%)	

*Smoking cigarettes*				
Yes	9 (28.1%)	6 (10.3%)	15 (16.7%)	*P* = 1.0000 (Fisher's exact test)
No	23 (71.9%)	52 (89.7%)	75 (83.3%)	

*Coffee*				
Yes	23 (71.9%)	46 (79.3%)	69 (76.7%)	*P* = 0.4451 (Fisher's exact test)
No	9 (28.1%)	12 (20.7%)	21 (23.3%)	

*Alcohol consumption*				
Yes	29 (90.6%)	51 (87.9%)	80 (88.9%)	*P* = 0.5321 (Fisher's exact test)
No	3 (9.4%)	7 (12.1%)	10 (11.1%)	

**Table 2:** Duration of pesticide use, storage and awareness on toxicity.

Question	Reference group	Pesticide users	Total	*P* value
*Duration of agro pesticides use*				
<1 year		10 (17.2%)		
From 1 to 5 years	/	20 (34.5%)		
From 6 to 10 years		13 (22.4%)		
>10 years		15 (25.9%)		

*Means/method used for elimination or disposal of empty pesticide containers*				
Throw in the field/farm		53 (91.4%)		
Throw in to river/stream		1 (1.7%)		
Bury in the ground		2 (3.4%)		
Burn		2 (3.4%)		

*Opinion about negative impacts of pesticide on self's health*				
Pesticides have no health effect	4 (12.5%)	13 (22.4%)	17 (18.9%)	*P* = 0.0022 (Chi-squared test)
Pesticides have minor effects	15 (46.9%)	38 (65.5%)	53 (58.9%)	
Pesticides have major effects	8 (25.0%)	6 (10.3%)	14 (15.6%)	
Pesticides are fatal	5 (15.6%)	1 (1.7%)	6 (6.7%)	

Place where purchased pesticides are stored				
At home		48 (82.8%)		
In the farm		10 (17.2%)		

*Place where a sprayer container is washed*				
In the field		56 (96.6%)		
In the river/irrigation canal		2 (3.4%)		

**Table 3:** Agro pesticide active ingredients used by farmers.

Chemical class	Active ingredient	Brand name(s)	Number (percentage) of farmers using pesticides	Frequency of the pesticide relative to all pesticides (%)
Fungicides (53.14%)	Mancozeb	Mancostar, agrizeb80, fongistar 72%WP	51 (87.9%)	15.37
	Metalaxyl	Callomil plus, nordox, ridomil plus, fongistar 72WP	14.7 (51.6%)	15.00
	Maneb	Trimangol 80WP	28 (48.3%)	5.19
	Copper oxide	Calomil plus	26 (44.8%)	4.81
	Copper sulphate	Kalarch	26 (44.8%)	4.81
	Benalaxyl	Galben plus	20 (34.5%)	3.70
	Carbendazim	Banko plus	8 (13.8%)	1.48
	Chlorothalonil	Banko plus	8 (13.8%)	1.48
	Tebuconazole	Folicure 250	7 (12.1%)	1.30

Insecticides (24.08%)	Imidacloprid	Gongfut 50EC, Parastar	26 (44.8%)	3.15
	Lindane	Gammalin 20	23 (39.7%)	4.26
	Ethoprophos	Mocap	22 (37.9%)	4.07
	Lambda cyahalothrine	Parastar, K optimal	20 (34.5%)	3.89
	Malathion	Poudrox	9 (15.5%)	1.67
	Thiamethoxam	Actara	9 (15.5%)	1.67
	Acetamipride	K optimal	8 (13%)	1.48
	Cypermethrin	Gongfut 50EC	8 (13.8%)	1.48
	Dimethoate	Dimex 40EC	7 (12.1%)	1.30
	Abamectin	Acarius	6 (10.3%)	1.11

Herbicides (22.78%)	Glyphosate	Glyphader, roundup	57 (98.3%)	12.04
	Clomazone	Command, commence	24 (41.4%)	4.63
	Glyphosate trimesium	Touchdown	13 (22.4%)	2.41
	Paraquat	Plantoxone super	13 (22.4%)	2.41
	Diuron	Diuralm 800SC	7 (12.1%)	1.30

## Data Availability

The biochemical and theoretical data used to support the findings of this study are restricted by the Institutional Ethics Committee for Human Research of the Faculty of Health Science of the University of Buea in Cameroon (Ref: 2016/0.53/UB/FHS/IRB) in order to protect participants privacy. Data are available from F.P.T. Manfo (faustinpascal@yahoo.fr), for researchers who meet the criteria for access to confidential data.

## Supplementary Materials

Supplementary Materials Supplement 1: ALT/AST ratio in a reference population and farmers using agropesticides. Supplement 2: sub-statistical analysis of the effect of agrochemicals active principles or formulations on ALT activity (U/L). Supplement 3: Creatinine/uric acid ratio in a reference population and farmers using agropesticides.
