# Supplementary material for: Evaluation of the Effects of Agro Pesticides Use on Liver and Kidney Function in Farmers from Buea, Cameroon
Source: J Toxicol. 2020 Jan 29;2020:2305764. doi: 10.1155/2020/2305764 (PMC7204329; doi:10.1155/2020/2305764)
Supplement: Supplementary Materials — Supplement 1: ALT/AST ratio in a reference population and farmers using agropesticides. Supplement 2: sub-statistical analysis of the effect of agrochemicals active principles or formulations on ALT activity (U/L). Supplement 3: Creatinine/uric acid ratio in a reference population and farmers using agropesticides. [file 2305764.f1.pdf]

SUPPLEMENTARY INFORMATION

Evaluation of the effects of agropesticides use on liver and kidney function in farmers from Buea, Cameroon

Faustin Pascal Tsagué Manfo[1]*, Sharon Asukia Mboe[1], Edouard Akono Nantia[2], Ferdinand Ngoula[3], Phélix Bruno Telefo[4], Paul Fewou Moundipa[5], Fidelis Cho-Ngwa[6]

[1] Department of Biochemistry and Molecular Biology, Faculty of Science, University of Buea, PO Box 63 Buea, Cameroon;

[2] Department of Biochemistry, Faculty of Science, University of Bamenda, PO Box 39 Bambili, Cameroon;

[3] Department of Animal Sciences, Faculty of Agronomy and Agricultural Sciences, University of Dschang, P.O. Box 188 Dschang, Cameroon;

[4] Department of Biochemistry, Laboratory of Medicinal Plant Biochemistry, Food Science, and Nutrition, Faculty of Science, University of Dschang, Dschang, Cameroon;

[5] Laboratory of Pharmacology and Toxicology, Department of Biochemistry, Faculty of Science, University of Yaoundé I, P.O. Box 812 Yaoundé, Cameroon

[6] Laboratory for Drugs and Molecular Diagnostics Research (ANDI Centre of Excellence for Onchocerciasis Drug Research), Biotechnology Unit, University of Buea, Cameroon;

* Correspondence should be addressed to F. P. T. Manfo; faustinpascal@yahoo.fr

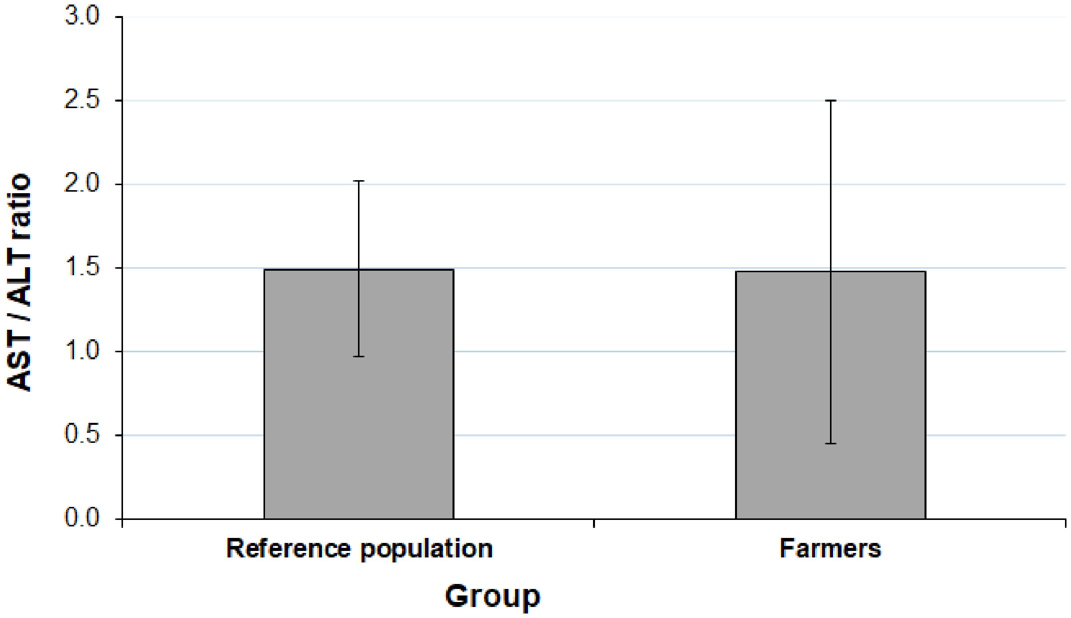

Supplement 1: ALT / AST ratio in a reference population and farmers using agropesticides.

ALT: Alanine aminotransferase; AST: Aspartate aminotransferase. No significant difference between the 2 study populations (P = 0.926; Welch t-test)

Supplement 2: sub-statistical analysis of the effect of agrochemicals active principles or formulations on ALT activity (U/L)

		Active principles			Pesticide formulations		
		Metalaxyl	Copper oxide	Ethoprophos	Callomil Plus	Mocap	Parastar
Non users	Mean	12.6	16.2	16.4	16.2	16.4	17.3
	SD	2.6	7.3	7.4	7.3	7.4	7.1
	n	7	32	35	32	35	45
Users	Mean	19.2	21.3	21.6	21.3	21.6	22.7
	SD	7.9	7.5	7.3	7.5	7.3	8.7
	n	50	25	22	25	22	12
P value (T-test)		*0.0326*	*0.0128*	*0.0123*	*0.0128*	*0.0123*	*0.0303*

"n" represents the sample size

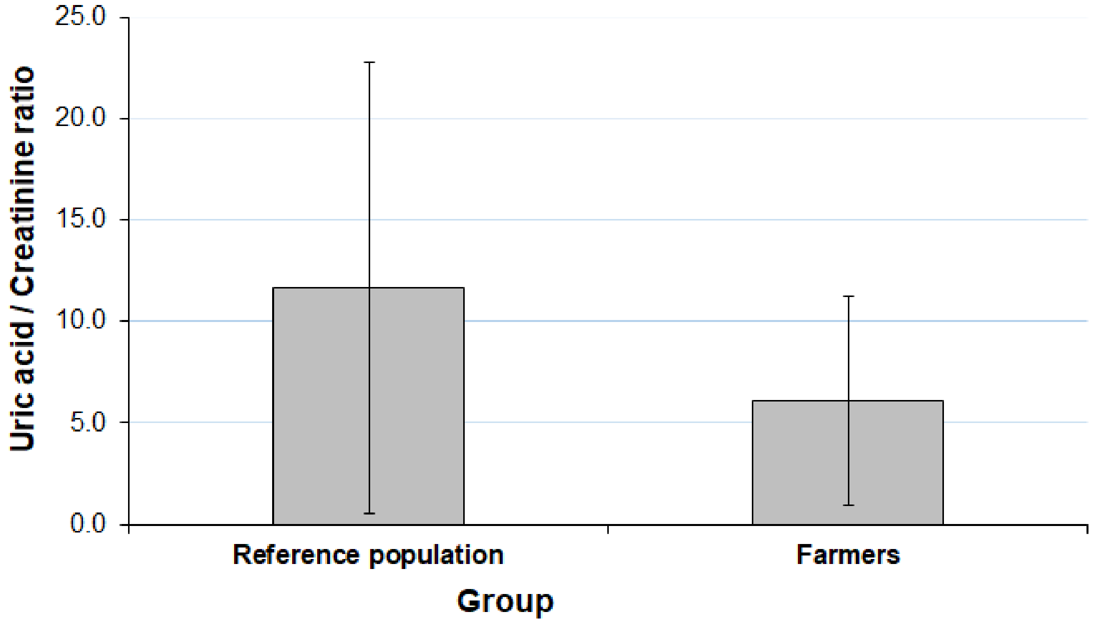

Supplement 3: Creatinine / uric acid ratio in a reference population and farmers using agropesticides.

No significant difference between the 2 study populations (P = 0.090; Welch t-test).
